# Incorporating alternative Polygenic Risk Scores into the BOADICEA breast cancer risk prediction model

**DOI:** 10.1158/1055-9965.EPI-22-0756

**Published:** 2023-01-13

**Authors:** Nasim Mavaddat, Lorenzo Ficorella, Tim Carver, Andrew Lee, Alex P. Cunningham, Michael Lush, Joe Dennis, Marc Tischkowitz, Kate Downes, Donglei Hu, Eric Hahnen, Rita K. Schmutzler, Tracy L. Stockley, Gregory S. Downs, Tong Zhang, Anna M. Chiarelli, Stig E. Bojesen, Cong Liu, Wendy K. Chung, Monica Pardo, Lidia Feliubadaló, Judith Balmaña, Jacques Simard, Antonis C. Antoniou, Douglas F. Easton

**Affiliations:** 1 Centre for Cancer Genetic Epidemiology, Department of Public Health and Primary Care, University of Cambridge, Cambridge, UK; 2 Department of Medical Genetics and National Institute for Health Research, Cambridge Biomedical Research Centre, The University of Cambridge, Cambridge, UK; 3 Cambridge Genomics Laboratory, Cambridge University Hospitals NHS Foundation Trust, Cambridge, UK; 4 Division of General Internal Medicine, Department of Medicine, University of California San Francisco, San Francisco, California, USA; 5 Center for Familial Breast and Ovarian Cancer, Faculty of Medicine and University Hospital Cologne, University of Cologne, Cologne, Germany; 6 Center for Integrated Oncology (CIO), Faculty of Medicine and University Hospital Cologne, University of Cologne, Cologne, Germany; 7 Center for Molecular Medicine Cologne (CMMC), Faculty of Medicine and University Hospital Cologne, University of Cologne, Cologne, Germany; 8 Advanced Molecular Diagnostics Laboratory, Princess Margaret Cancer Centre, Toronto, Ontario, Canada; 9 Department of Laboratory Medicine and Pathobiology, The University of Toronto, Ontario, Canada; 10 Division of Clinical Laboratory Genetics, Laboratory Medicine Program, University Health Network, Toronto, Canada; 11 Dalla Lana School of Public Health, University of Toronto, Toronto, ON M5S 1A1, Canada; 12 Ontario Health, Cancer Care Ontario, Toronto, ON M5G 2L3, Canada; 13 Copenhagen General Population Study, Herlev and Gentofte Hospital, Copenhagen University Hospital, Herlev, Denmark; 14 Department of Clinical Biochemistry, Herlev and Gentofte Hospital, Copenhagen University Hospital, Herlev, Denmark; 15 Faculty of Health and Medical Sciences, University of Copenhagen, Copenhagen, Denmark; 16 Department of Biomedical Informatics, Columbia University Irving Medical Center, New York, New York, USA; 17 Departments of Pediatrics and Medicine, Columbia University, New York, NY, USA; 18 Hereditary Cancer Genetics Group, Vall d’Hebron Institut d’Oncologia, Barcelona, Spain; 19 Hereditary Cancer Program, Catalan Institute of Oncology (ICO), L’Hospitalet de Llobregat, Spain; 20 Program in Molecular Mechanisms and Experimental Therapy in Oncology (Oncobell), IDIBELL, L’Hospitalet de Llobregat, Spain; 21 Centro de Investigación Biomédica en Red de Cáncer (CIBERONC), Madrid, Spain; 22 Medical Oncology Department, University Hospital of Vall d’Hebron, Barcelona, Spain; 23 Department of Molecular Medicine, Université Laval and CHU de Québec-Université Laval Research Center; 24 Centre for Cancer Genetic Epidemiology, Department of Oncology, University of Cambridge, Cambridge, UK

**Keywords:** cancer, breast, risk, prediction, polygenic

## Abstract

**Background:**

The multifactorial risk prediction model BOADICEA enables identification of women at higher or lower risk of developing breast cancer. BOADICEA models genetic susceptibility in terms of the effects of rare variants in breast cancer susceptibility genes and a polygenic component, decomposed into an unmeasured and a measured component - the polygenic risk score (PRS). The current version was developed using a 313 SNP PRS. Here, we evaluated approaches to incorporating this PRS and alternative PRS in BOADICEA.

**Methods:**

The mean, standard deviation (SD), and proportion of the overall polygenic component explained by the PRS (α^2^) need to be estimated. *α* was estimated using logistic regression, where the age-specific log-odds ratio is constrained to be a function of the age-dependent polygenic relative risk in BOADICEA; and using a retrospective likelihood (RL) approach that models, in addition, the unmeasured polygenic component.

**Results:**

Parameters were computed for 11 PRS, including 6 variations of the 313 SNP PRS used in clinical trials and implementation studies. The logistic regression approach underestimates *α,* as compared with the RL estimates. The RL *α* estimates were very close to those obtained by assuming proportionality to the odds ratio per 1 SD, with the constant of proportionality estimated using the 313 SNP PRS. Small variations in the SNPs included in the PRS can lead to large differences in the mean.

**Conclusions:**

BOADICEA can be readily adapted to different PRS in a manner that maintains consistency of the model.

**Impact:**

The methods described facilitate comprehensive breast cancer risk assessment.

## Introduction

BOADICEA^1,2^ is a risk prediction algorithm for predicting breast and ovarian cancer risk on the basis of genetic and non-genetic factors. The algorithm incorporates the effects of common genetic variants, summarised in a polygenic risk score (PRS), in addition to the effects of pathogenic variants in major breast cancer susceptibility genes, other lifestyle/hormonal risk factors and cancer family history.

The current version (v6)^1,2^ has been specifically developed to incorporate the 313 SNP PRS of Mavaddat *et al*.^3^; this PRS was developed using the very large data set of the Breast Cancer Association Consortium (BCAC) and extensively validated in prospective studies. However, as larger genome-wide association studies (GWAS) and novel statistical methods become available, new PRS are being continually developed. In addition, PRS developed for clinical translation and generated in different health care systems use a variety of technologies, including both targeted sequencing panels and genotyping arrays, and surrogate SNPs are often required. The BOADICEA algorithm itself is flexible and can incorporate any PRS for which the relevant parameters are known. These parameters are the mean (μ) and standard deviation (σ) of the PRS in the population, and the proportion (*α*^2^) of the polygenic variance attributable to the PRS. In practice, the PRS can be normalised and supplied as a Z-score, in which case only parameter *α* is required. By modelling the PRS as the proportion of a (fixed) polygenic component, the predicted familial risks remain consistent, irrespective of the PRS used, and importantly there is no double counting of the effect of the PRS and cancer family history.

Here, we discuss the incorporation of alternative PRSs into BOADICEA, and provide the relevant parameters for a number of PRS that have been developed, including several that are in use in clinical applications.

## Materials and Methods

BOADICEA models breast cancer risks such that the incidence of breast cancer at age t is of the form^1,4^:

(1) $$\lambda \left(t\right)=\lambda_{0}\left(t\right)exp\left(\delta_{g\left(i\right)}\left(t\right)+\sigma_{P}\left(t\right)x_{P}^{\left(i\right)}+\Sigma_{\rho }\beta_{\rho }z_{\rho i}\right)$$

Here *λ*_0_(*t*) is the baseline incidence. The term *δ*_*g*(*i*)_ (*t*) models the major gene component for individual i (*δ_k_*(*t*) being the age-specific log-hazard ratio associated with genotype *k*). $\sigma_{P}\left(t\right)x_{P}^{\left(i\right)}$ models the polygenic component, *σ_P_*(*t*) being the polygenic standard deviation and $x_{P}^{\left(i\right)}$ the normalised polygenic component for individual i. The final term models the effects of other risk factors. The polygenic variance, $\sigma_{P}^{2}\left(t\right)$ is allowed to be age-dependent and assumed to be a linear function of age t:

$$\sigma_{P}^{2}\left(t\right)=\gamma +\theta t$$

The parameters *γ* and *θ* have been previously estimated, using complex segregation analysis, as 4.86 and -0.06 respectively^4^.

The PRS is incorporated into BOADICEA by partitioning the total polygenic component *x_P_* into the sum of a known component *x_K_* measured by the PRS, and an unmeasured residual component *x*_*R*_^1^. The variance due to the known component is of the form^3^:

(2) $$\sigma_{K}^{2}\left(t\right)=\alpha^{2}\left(\gamma +\theta t\right)$$

*σ_K_*(*t*) can also be interpreted as the age-specific log-hazard ratio per unit SD of the PRS, conditional on other risk factors. Note that in Mavaddat *et al*.^3^ equation (2) is written $\sigma_{K}^{2}\left(t\right)=\gamma^{2}\left(\alpha +\beta t\right)$. The change of symbols is for consistency with Lee *et al*.^1^ and the Canrisk platform (www.canrisk.org), where the proportion of the polygenic variance explained by the PRS is denoted as *α*^2^.

### Estimation of a and incorporating alternative PRS

The key parameter is *a.* The first approach to estimating this parameter makes the simplifying assumption that the polygenic standard deviation of the known polygenic component in BOADICEA, *σ_K_*(*t*) can be approximated by the marginal age-specific log-hazard ratio per unit SD of the PRS^3^ (see Supplementary methods). This can then be estimated using cohort data or (approximately, making the rare disease assumption) case-control data, by first transforming the PRS using:

(3) $$S^{\prime }=x_{K}\sqrt{\gamma +\theta \text{t}}$$

where *x_K_* is the standardised (per unit SD) version of the proposed PRS. *S′* is then included as a covariate in a Cox or logistic regression model: the parameter (log-hazard or log-odds ratio) estimate corresponding to the covariate *S′* gives the required *α* parameter, which we denote *α*_GLM_. This method was applied to 22,767 controls and 16,151 women diagnosed with invasive breast cancer from the validation and prospective test sets used in Mavaddat et al^3^ (Supplementary Tables S1 and S2). The analysis was restricted to women of European ancestry with age of diagnosis or last observation less than 80 years (after application of inclusion/exclusion criteria, mean age at diagnosis = 59.9 (sd=10) years for cases, and 57.1 (sd=10.4) years for controls). Analyses were adjusted for country in which the study was conducted (15 countries) and 10 Principal Components (PCs).

The above analyses make the simplifying assumption that the marginal PRS effect size is a good approximation to the effect size conditional on other risk factors. This is likely to be a reasonable assumption for non-genetic risk factors, which have relatively small effects on risk and appear to be independent of the PRS, as shown in recent analyses of the combined effect of breast cancer PRS and individual SNPs with life-style/environmental risk factors including questionnaire-based factors^5–10^. However, it may not be true for other genetic factors, in particular the unmeasured polygenic component. Although the PRS and the residual polygenic component are assumed to be conditionally independent, individuals with a high polygenic component are more likely to develop the disease at an early age. This results in a negative correlation between the PRS and the residual polygenic component at later ages, which leads to an underestimation of the PRS effect size if the latter is not allowed for. To address this problem, we also estimated *α* using a retrospective likelihood approach (*α_RL_*), applied to the same BCAC data set. In this analysis, the observed PRS is computed conditional on the phenotypes of the individuals (age of diagnosis and case/control status), explicitly allowing for the unmeasured polygenic component. Details are given in the Supplementary Methods. This approach requires overall population age-specific incidence rates to be specified. For this purpose, the rates for England and Wales 2016-2018 were used (https://www.cancerresearchuk.org/health-professional/cancer-statistics/incidence/age).

Since the mean PRS varies by country, we first regressed the PRS on country and principal components, adjusted for case/control status, and performed the analyses on the residual PRS. The likelihood was maximised using the optimize function in R. 95% Confidence Intervals were obtained using a grid of values for *α_RL_*, and finding the difference between the log-likelihoods and the maximum log-likelihood.

As a third approach, we derived an approximate estimate *α* from the log-odds ratio per unit SD (η), by calibrating against PRS313 as a standard. From equations (1) and (2) in the methods above it can be seen that, under the rare disease assumption, the marginal hazard ratio associated with the PRS should approximate the conditional hazard ratio. If differential age effects can also be ignored, *α* should therefore be approximately proportional to η. This allows *α* to be estimated using PRS313 as a standard. Thus: $\alpha_{APP}=\frac{\text{η}}{{\text{η}}_{0}}\alpha_{0}$ where η_0_ and *α*_0_ are the corresponding estimates for PRS313. This provides a simple method that could be applied to PRS developed and validated on a different data set.

We computed the relevant parameters for PRS313 and 10 additional PRS (Supplementary Tables S3 and S4; SNP positions based on Genome Reference Consortium Human Build 37 (GRCh37)). PRS313 includes two variants (22_29203724_C_T and 22_29551872_A_G) which are correlated with the protein truncating variant CHEK2*1100delC, and some of the derivative PRS also include these SNPs. This could result in overestimation of risk in CHEK2*1100delC carriers if the PRS is used in conjunction with gene-panel testing, because BOADICEA assumes that the PRS and major gene genotypes are independent in the population. We therefore also considered PRS without these variants. (Note that *CHEK2* p.I157T (22_29121087_A_G) is also included in PRS313 but is only weakly correlated with CHEK2*1100delC and does not introduce a bias). The means and standard deviations of each PRS, and the proportion (α) of the polygenic variance attributable to these alternative PRS were derived in the same data set (Supplementary Tables S1 and S2), namely the validation and prospective sets described by Mavaddat *et al*.^3^.

All studies included in this analysis were approved by the relevant local ethical review boards and used appropriate consent procedures. SEARCH was approved by the NRES Committee East of England - Cambridge South.

## Results

### PRS Examples

Eleven alternative PRS were constructed. Six of these are modifications of the PRS313, designed for clinical implementation. The BRIDGES PRS was developed as an NGS panel test to facilitate clinical translational studies of BOADICEA implemented in the context of genetic testing of women with a family history (https://bridges-research.eu/). Of 313 variants, 295 could be designed and a further 11 were replaced by surrogate markers (r^2^>0.9 in Europeans). The PERSPECTIVE I&I PRS was designed to facilitate risk stratified screening in the context of population-based mammographic screening in Ontario and Quebec^11^. This PRS was designed as an NGS panel: 287 of 313 markers could be designed and a further 8 were surrogates. The EastGLH PRS was designed by the NHS East Genomic Laboratory Hub for use in a randomised control trial of women testing positive for an inherited pathogenic/likely pathogenic gene variant in *BRCA1, BRCA2, PALB2, CHEK2* or *ATM,* using a NGS panel of 303 markers^12^. The PRISMA PRS, designed as genotyping array of 268 markers (37 surrogates), was developed to provide multifactorial cancer risks to women attending genetic clinics in Spain. The eMERGE PRS consisted of 308 markers and is part of a large US study aiming to communicate PRS-based genome-informed risk assessment across multiple diseases (https://emerge-network.org). DBDS299, using data from the Danish Blood Donor Study (https://bmjopen.bmj.com/content/9/6/e028401) is used in a research study to validate BOADICEA in the Danish population. In addition, we included the earlier PRS77 developed using BCAC data and comprising genome-wide significant SNPs, PRS3820 developed by Mavaddat *et al*.^3^ using Lasso penalised regression, and two PRS (WISDOM75 and WISDOM120) developed for the WISDOM clinical trial^13^ (Clinical Trials identifier NCT02620852). We also considered all of the above PRS without 22_29203724_C_T and 22_29551872_A_G, SNPs correlated with CHEK2*1100delC, as described in the Methods.

### PRS Parameters

Table 1 summarises the estimated parameters for PRS313 and each of the alternative PRS. As expected, the 6 PRS that are variations on PRS313 have very similar effect sizes, expressed as log-OR per 1 SD, reflecting the fact that only a few variants are not accounted for. The *α_RL_* parameters for these 6 PRS are also similar, and only marginally lower than PRS313 estimate (0.441, 95%CI 0.430-0.445). The effect sizes for PRS77 (both in terms of the log-OR per 1 SD and α) were smaller than for PRS313, while PRS3820 had larger effect sizes. The two WISDOM and PRISMA PRSs also had somewhat smaller effect sizes than PRS313. Removal of the 2 chromosome 22 SNPs had only a small effect on the estimated log-OR per 1SD, and α – for example reducing *α_RL_* from 0.441 to 0.439 for PRS313. The α values computed using the simpler logistic regression approach (*α*_GLM_) were smaller than those generated using the retrospective likelihood approach for all PRS.

**Figure 1:**
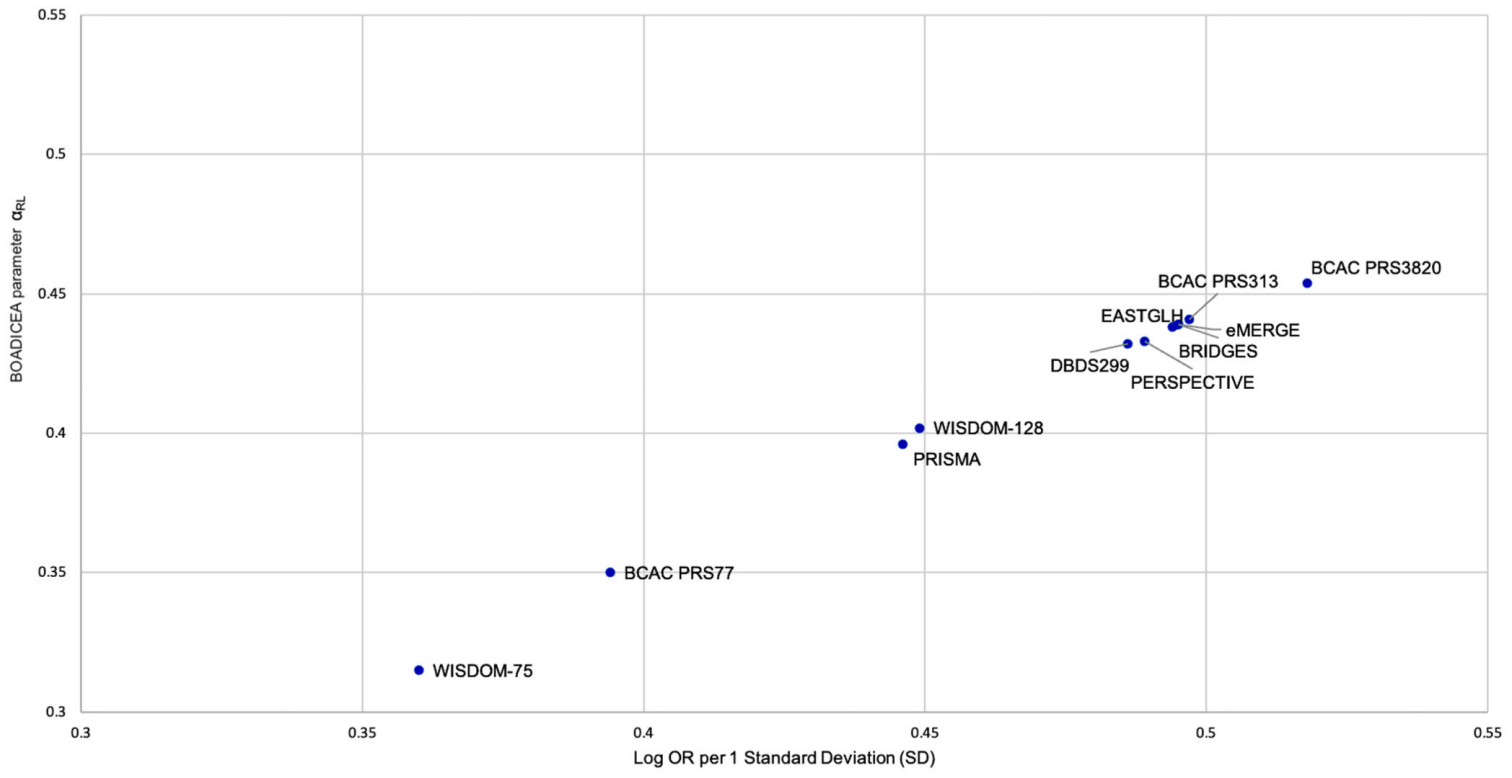
Graph of BOADICEA parameter alpha (α_RL_) vs log OR per 1 SD Graph of BOADICEA parameter alpha (α_RL_) vs log OR per 1 SD for PRS313 and alternative PRS. α_RL_ were estimated using the retrospective likelihood method as described in the Methods and Supplementary Methods.

We note that the *α_RL_* are approximately proportional to the PRS effect sizes, expressed as odds-ratio per 1 SD (Table 1; Figure 1). Using the PRS313 as the standard, the predicted *α* value assuming proportionality is given by *α_APP_* = 0.887*η* (Table 1; Figure 1). These predicted values were very similar to the *α_RL_* values for all PRS.

## Discussion

We evaluated approaches to incorporating alternate breast cancer PRSs into the risk prediction algorithm BOADICEA. The α values computed using the simpler logistic regression approach (α_GLM_) were consistently smaller than those generated using the retrospective likelihood approach (α_RL_), for all PRS. This difference can be explained by the fact that the logistic regression approach does not account for the residual component. Women with a high polygenic component are more likely to develop the disease at an early age, resulting in a negative correlation between the PRS and the residual polygenic component, which leads to an underestimation of the PRS effect size if the latter is not allowed for, a phenomenon related to index event bias^14^.

We showed further that the α parameters derived from the log-odds ratio estimate by assuming proportionality were very close to the α_RL_ estimates. This suggests that this approach is likely to be reasonably accurate for other PRS, at least across the range of effect sizes considered here, providing a very straightforward approach to incorporating a PRS developed on another data set if a log-odds ratio estimate is already available.

A striking observation is the very large difference in the means of the different PRS. This reflects the fact that the removal of a few SNPs with important weights can have a substantial effect on the mean. For example, the means for the PRS excluding the chromosome 22 SNPs are higher. While the mean has no intrinsic significance, this emphasises the importance of correctly normalising the PRS. In particular, because BOADICEA also incorporates the effects of *CHEK2* protein truncating variants, we recommend using the PRS without these SNPs when gene-panel testing is performed.

It is important to note that estimates derived from European ancestry populations may not be applicable to individuals of other ancestries. The effect sizes may differ among populations, for example due to differences in linkage disequilibrium structure. This has been shown for PRS313, for which somewhat smaller effect sizes have been estimated in Asian and African-American populations^15–18^. In addition, the mean PRS can vary significantly by population –PRS313 has a higher mean in both Asian and African-American populations than in Europeans. This again emphasises the importance of calibrating the PRS to the relevant population distribution. The argument that the retrospective likelihood approach is preferable and provides a more reliable estimate of α should also hold in non-European populations.

The analyses used here adjusted the PRS for both the country in which the study was conducted and ancestry informative principal components. An adjustment is necessary since the mean PRS varies by country, even among European populations (and this is not reflected in differences in incidence rates). However, it is possible that adjustment for both country and principal components is over-conservative. Further analyses in large population-specific data sets may be able to address this.

The approaches described allow BOADICEA to be adapted for use with any PRS in a consistent manner. However, it should be emphasised that the main validations of BOADICEA utilised PRS313^19–23^. For PRS that are substantially different, and particularly as more informative PRS are generated through larger GWAS, further prospective validation in independent external data-sets will be required. We also note that the current formulation of BOADICEA assumes that the age-specific effects of the PRS and the residual polygenic component (as measured by the log-hazard ratio per 1 SD) are proportional. This significantly simplifies the algorithm, but it is possible that better predictions may be available by allowing differential age-specific effects.

The BOADICEA algorithm has been extensively validated, particularly when incorporating PRS313^19–23^ in addition to other risk factors. It is available through the CanRisk (www.canrisk.org) tool^24^ and is widely used in the context of women with cancer family history or undergoing gene-panel testing, including several ongoing clinical implementation studies. The CanRisk tool provides the facility to incorporate a PRS as a Z-score, providing that the α parameter is known. The methods described here allow other PRSs to be used with BOADICEA via CanRisk and hence facilitate more widespread comprehensive breast cancer risk assessment.

## Supplementary Material

Supplementary methods

Table S1

Table S2

Table S3

Table S4

## Figures and Tables

**Table 1:** Summary parameters for alternative PRSs^a^

	No of SNPs	Mean controls	Mean cases	SD controls	SD cases	Log OR per 1 SD	95%CI	α_GLM^b^_	95%CI	α_RL^c^_	95%CI	Ratio (α_RL_:Log OR per 1 SD)	Predicted α_RL_
**BCAC PRS313**	313	-0.424	-0.114	0.611	0.619	0.497	0.476 - 0.519	0.397	0.378 - 0.415	0.441	0.430 - 0.455	0.887	0.441
**BRIDGES**	306	-0.422	-0.114	0.608	0.615	0.495	0.474 - 0.517	0.394	0.376 - 0.413	0.439	0.425 - 0.450	0.886	0.439
**PERSPECTIVE**	295	-0.448	-0.147	0.599	0.609	0.489	0.468 - 0.511	0.389	0.371 - 0.408	0.433	0.420 - 0.450	0.886	0.434
**EASTGLH**	303	-0.407	-0.100	0.606	0.613	0.494	0.472 - 0.516	0.394	0.375 - 0.412	0.438	0.420 - 0.450	0.886	0.438
**PRISMA**	268	0.322	0.575	0.558	0.565	0.446	0.424 - 0.467	0.358	0.339 - 0.376	0.396	0.385 - 0.410	0.890	0.395
**eMERGE**	308	-0.456	-0.150	0.608	0.616	0.495	0.473 - 0.516	0.394	0.376 - 0.413	0.439	0.425 - 0.450	0.887	0.439
**DBDS299**	299	-0.508	-0.211	0.596	0.605	0.486	0.465 - 0.508	0.387	0.369 - 0.406	0.432	0.440 - 0.455	0.888	0.431
**BCAC PRS77**	77	-0.892	-0.703	0.449	0.460	0.394	0.373 - 0.415	0.310	0.292 - 0.328	0.350	0.340 - 0.360	0.889	0.349
**BCAC PRS3820**	3820	-0.445	-0.199	0.460	0.463	0.518	0.496 - 0.540	0.412	0.393 - 0.431	0.454	0.440 - 0.470	0.878	0.459
**WISDOM75**	74	-1.057	-0.835	0.567	0.573	0.360	0.338 - 0.381	0.281	0.263 - 0.299	0.315	0.300 - 0.330	0.875	0.319
**WISDOM128**	126	-0.180	0.041	0.464	0.478	0.449	0.427 - 0.470	0.355	0.337 - 0.374	0.402	0.390 - 0.415	0.895	0.398
PRS constructed as above but excluding SNPs correlated with CHEK2*1100delC													
**BCAC PRS311**	311	-0.092	0.217	0.609	0.618	0.496	0.474 - 0.518	0.395	0.377 - 0.414	0.439	0.425 - 0.455	0.886	0.440
**BRIDGES**	305	-0.428	-0.121	0.607	0.615	0.495	0.473 - 0.516	0.394	0.375 - 0.412	0.438	0.425 - 0.450	0.886	0.439
**PERSPECTIVE**	293	-0.116	0.183	0.598	0.607	0.488	0.466 - 0.510	0.388	0.369 - 0.406	0.432	0.420 - 0.445	0.885	0.433
**EASTGLH**	301	-0.075	0.230	0.604	0.612	0.492	0.471 - 0.514	0.392	0.374 - 0.411	0.436	0.420 - 0.450	0.885	0.437
**eMERGE**	306	-0.124	0.181	0.606	0.614	0.493	0.471 - 0.515	0.393	0.374 - 0.411	0.437	0.425 - 0.450	0.886	0.437
**DBDS299**	297	-0.177	0.119	0.594	0.603	0.485	0.463 - 0.506	0.386	0.367 - 0.404	0.430		0.887	0.430
**BCAC PRS3818**	3818	-0.227	0.018	0.459	0.462	0.517	0.495 - 0.539	0.411	0.392 - 0.430	0.453	0.440 - 0.465	0.877	0.458

SNP, single nucleotide polymorphism; SD, standard deviation, OR; Odds Ratio; CI, Confidence Interval; GLM, Generalized Linear Model; RL, Retrospective Likelihood

a Results based on 22767 controls and 16151 cases in the prospective and validation sets from Mavaddat et al. European women with known age and age less than 80;

b α_GLM_ is based on logistic regression adjusted for country in which studies were conducted and 10 principal components.

c α_RL_ is based on retrospective likelihood method as described in the Methods and Supplementary Methods.

BRIDGES includes 22_29203724_C_T but not 22_29551872_A_G. BCAC PRS313, BCAC 3820, eMERGE, DBDS299, EASTGLH and PERSPECTIVE include both 22_29203724_C_T and 22_29551872_A_G. PRISMA has neither 22_29551872_A_G nor 22_29203724_C_T and includes 37 surrogates

## Data Availability

Data were generated by the authors and available on request.

## References

[1] Lee A, Mavaddat N, Wilcox AN, Cunningham AP, Carver T, Hartley S (2019). BOADICEA: a comprehensive breast cancer risk prediction model
incorporating genetic and nongenetic risk factors. Genet Med.

[2] Lee A, Mavaddat N, Cunningham A, Carver T, Ficorella L, Archer S (2022). Enhancing the BOADICEA cancer risk prediction model to
incorporate new data on RAD51C, RAD51D, BARD1, updates to tumour pathology
and cancer incidence. J Medical Genet.

[3] Mavaddat N, Michailidou K, Dennis J, Lush M, Fachal L, Lee A (2019). Polygenic Risk Scores for Prediction of Breast Cancer and Breast
Cancer Subtypes. Am J Hum Genet.

[4] Antoniou AC, Cunningham AP, Peto J, Evans DG, Lalloo F, Narod SA (2008). The BOADICEA model of genetic susceptibility to breast and
ovarian cancers: updates and extensions. Br J Cancer.

[5] Kapoor PM, Mavaddat N, Choudhury PP, Wilcox AN, Lindström S, Behrens S (2021). Combined Associations of a Polygenic Risk Score and Classical
Risk Factors With Breast Cancer Risk. J Natl Cancer Inst.

[6] Rudolph A, Song M, Brook MN, Milne RL, Mavaddat N, Michailidou K (2018). Joint associations of a polygenic risk score and environmental
risk factors for breast cancer in the Breast Cancer Association
Consortium. Int J Epidemiol.

[7] Barrdahl M, Canzian F, Joshi AD, Travis RC, Chang-Claude J, Auer PL (2014). Post-GWAS gene-environment interplay in breast cancer: results
from the Breast and Prostate Cancer Cohort Consortium and a meta-analysis on
79,000 women. Hum Mol Genet.

[8] Travis RC, Reeves GK, Green J, Bull D, Tipper SJ, Baker K (2010). Gene-environment interactions in 7610 women with breast cancer:
prospective evidence from the Million Women Study. Lancet.

[9] Kapoor PM, Lindström S, Behrens S, Wang X, Michailidou K, Bolla MK (2020). Assessment of interactions between 205 breast cancer
susceptibility loci and 13 established risk factors in relation to breast
cancer risk, in the Breast Cancer Association Consortium. Int J Epidemiol.

[10] Wang X, Chen H, Kapoor PM, Su Y-R, Bolla MK, Dennis J (2022). A Genome-Wide Gene-Based Gene-Environment Interaction Study of
Breast Cancer in More than 90,000 Women. Cancer Research Comm.

[11] Brooks JD, Nabi HH, Andrulis IL, Antoniou AC, Chiquette J, Després P (2021). Personalized Risk Assessment for Prevention and Early Detection
of Breast Cancer: Integration and Implementation (PERSPECTIVE
I&I). JPers Med.

[12] Archer S, Fennell N, Colvin E, Laquindanum R, Mills M, Dennis R (2022). Personalised Risk Prediction in Hereditary Breast and Ovarian
Cancer: A Protocol for a Multi-Centre Randomised Controlled
Trial. Cancers (Basel).

[13] Shieh Y, Eklund M, Madlensky L, Sawyer SD, Thompson CK, Stover Fiscalini A (2017). Breast Cancer Screening in the Precision Medicine Era: Risk-Based
Screening in a Population-Based Trial. J Natl Cancer Inst.

[14] Dudbridge F, Allen RJ, Sheehan NA, Schmidt AF, Lee JC, Jenkins RG (2019). Adjustment for index event bias in genome-wide association
studies of subsequent events. Nat Commun.

[15] Fritsche LG, Ma Y, Zhang D, Salvatore M, Lee S, Zhou X (2021). On cross-ancestry cancer polygenic risk scores. PLoS Genet.

[16] Du Z, Gao G, Adedokun B, Ahearn T, Lunetta KL, Zirpoli G (2021). Evaluating Polygenic Risk Scores for Breast Cancer in Women of
African Ancestry. J Natl Cancer Inst.

[17] Liu C, Zeinomar N, Chung WK, Kiryluk K, Gharavi AG, Hripcsak G (2021). Generalizability of Polygenic Risk Scores for Breast Cancer Among
Women With European, African, and Latinx Ancestry. JAMA Netw Open.

[18] Ho W-K, Tai M-C, Dennis J, Tai MC, Mariapun S, Li J (2022). Polygenic risk scores for prediction of breast cancer risk in
Asian populations. Genet Med.

[19] Li SX, Milne RL, Nguyen-Dumont T, Wang X, English DR, Giles GG (2021). Prospective Evaluation of the Addition of Polygenic Risk Scores
to Breast Cancer Risk Models. JNCI Cancer Spectr.

[20] Lakeman IMM, Rodríguez-Girondo M, Lee A, Ruiter R, Stricker BH, Wijnant SRA (2020). Validation of the BOADICEA model and a 313-variant polygenic risk
score for breast cancer risk prediction in a Dutch prospective
cohort. Genet Med.

[21] Pal Choudhury P, Brook MN, Hurson AN, Lee A, Mulder CV, Coulson P (2021). Comparative validation of the BOADICEA and Tyrer-Cuzick breast
cancer risk models incorporating classical risk factors and polygenic risk
in a population-based prospective cohort of women of European
ancestry. Breast cancer Res.

[22] Hurson AN, Pal Choudhury P, Gao C, Hüsing A, Eriksson M, Shi M (2021). Prospective evaluation of a breast-cancer risk model integrating
classical risk factors and polygenic risk in 15 cohorts from six
countries. Int J Epidemiol.

[23] Yang X, Eriksson M, Czene K, Lee A, Leslie G, Lush M (2022). Prospective validation of the BOADICEA multifactorial breast
cancer risk prediction model in a large prospective cohort
study. J Med Genetics.

[24] Carver T, Hartley S, Lee A, Cunningham AP, Archer S, Babb de Villiers C (2021). CanRisk Tool-A Web Interface for the Prediction of Breast and
Ovarian Cancer Risk and the Likelihood of Carrying Genetic Pathogenic
Variants. Cancer Epidemiol Biomarkers Prev.

