# Correction: Incorporating Alternative Polygenic Risk Scores into the BOADICEA Breast Cancer Risk Prediction Model

**DOI:** 10.1158/1055-9965.EPI-24-1661

**Published:** 2025-01-09

**Authors:** Nasim Mavaddat, Lorenzo Ficorella, Tim Carver, Andrew Lee, Alex P. Cunningham, Michael Lush, Joe Dennis, Marc Tischkowitz, Kate Downes, Donglei Hu, Eric Hahnen, Rita K. Schmutzler, Tracy L. Stockley, Gregory S. Downs, Tong Zhang, Anna M. Chiarelli, Stig E. Bojesen, Cong Liu, Wendy K. Chung, Monica Pardo, Lidia Feliubadaló, Judith Balmaña, Jacques Simard, Antonis C. Antoniou, Douglas F. Easton

In the original version of this article (1), there was an error in the code for calculating some of the values in Table 1. Specifically, the error related to values for the parameter alphaRL in the table, and the corresponding 95% confidence limits. After correcting this error, the new estimates are slightly larger than those previously reported. The error has been corrected in the latest online HTML and PDF versions of the article. The authors apologise for this error.
