# Supplementary material for: Incorporating alternative Polygenic Risk Scores into the BOADICEA breast cancer risk prediction model
Source: Cancer Epidemiol Biomarkers Prev. Author manuscript; Available in PMC 2023 Mar 7. (PMC9986688; doi:10.1158/1055-9965.EPI-22-0756)
Supplement: Supplementary methods [file EMS162586-supplement-Supplementary_methods.docx]

**Supplementary Methods**

**Incorporating alternative Polygenic Risk Scores into the BOADICEA breast cancer risk prediction model**

**Estimating parameters using a retrospective likelihood approach**

To implement the retrospective likelihood approach, we consider a simplified version of the BOADICEA model including just the polygenic component:

$$\lambda\left( t;x_{P} \right)=\lambda_{0}\left( t \right)exp(\sigma_{P}\left( t \right)x_{P})$$

Where $\lambda\left( t;x_{P} \right)$is the incidence at age *t* for an individual with standardised polygenotype $x_{P}$,$\lambda_{0}\left( t \right)$ is the baseline incidence and $\sigma_{P}^{2}\left( t \right)$ is the overall age-specific polygenic variance. In the current version of BOADICEA (v6) $\sigma_{P}^{2}\left( t \right)$ is assumed to vary linearly with age, i.e. $\sigma_{P}^{2}\left( t \right)=\gamma+\theta t$. For individual *j* with disease status *d_j_* =0 or 1 at age *t_i_,* the likelihood of the PRS given the phenotypes is:

$$L\left( \alpha\right)=\frac{\int\exp\left( -\Lambda(t_{j};\alpha x_{K}^{\left( j \right)}+\sqrt{1-\alpha^{2}}x_{U}) \right){\lambda_{0}(t_{j})}^{d_{j}}\exp\left( d_{j}\sigma_{P}\left( t_{j} \right)(\alpha x_{K}^{\left( j \right)}+\sqrt{1-\alpha^{2}}x_{U}) \right)\phi\left( x_{U} \right)dx_{U}}{\int\exp\left( -\Lambda(t_{j};x) \right)\exp\left( d_{j}\sigma_{P}\left( t_{j} \right)x \right)\phi(x)dx}$$

Where $x_{K}^{\left( j \right)}$ is the normalised PRS for individual *j*, $x_{U}$ denotes the unmeasured residual polygenic component, $\phi()$ is the standard normal density and

$\Lambda\left( t;x \right)=\int_{0}^{t} \lambda_{0}(u)exp(\sigma_{P}\left( u \right)x)du$. $\lambda_{0}(t)$ is fixed by the overall model (and does not depend on $\alpha$) such that the overall incidence rates, integrated over genotypes, agree with the population rates:

$$\frac{\int\exp\left( -\Lambda(t;x) \right)\lambda_{0}(t)exp \left( \sigma_{P}\left( t \right)x \right)\phi\left( x \right)dx}{\int\exp\left( -\Lambda(t;x) \right)exp \left( \sigma_{P}\left( t \right)x \right)\phi\left( x \right)dx}=i(t)$$

We also note that the denominator is fixed once the overall polygenic model is defined and does not depend on $\alpha$, so that the retrospective likelihood to be maximised is of the form:

$$l\left( \alpha\right)=const+\sum_{j} log\left( \int\exp\left( -\Lambda(t;\alpha x_{K}^{\left( j \right)}+\sqrt{1-\alpha^{2}}x_{U}) \right)\exp\left( d_{i}\sigma_{P}\left( t \right)(\alpha x_{K}^{\left( j \right)}+\sqrt{1-\alpha^{2}}x_{U}) \right)\phi\left( x_{U} \right)dx_{U} \right)$$

Or

$$l\left( \alpha\right)=const+\sum_{j} log\left( \int\exp\left( -\Lambda(t;\alpha x_{K}^{\left( j \right)}+\sqrt{1-\alpha^{2}}x_{U}) \right)\exp\left( d_{i}\sigma_{P}\left( t \right)x \right)\phi\left( \frac{x-\alpha x_{K}^{(j)}}{\sqrt{1-\alpha^{2}}} \right)dx \right)-Jlog(\sqrt{1-\alpha^{2}})$$

Where *J* is the total number of individuals. This is can be maximised numerically. We first compute $\Lambda_{0}\left( t \right)$ and the survivor functions $\exp\left( -\Lambda(t;\alpha x_{K}^{\left( j \right)}+\sqrt{1-\alpha^{2}}x_{U}) \right)$ for a dense grid of values of x, by iterations starting at age 0. The function $l\left( \alpha\right)$ is then maximised using *optimize* in R.

**Approximating the age-dependent polygenic standard deviation by the marginal age-specific log-hazard ratio per SD of the PRS.**

The marginal age-specific hazard at age *t* for an individual with PRS $x_{K}$ given by:

$$\frac{\int_{x_{U}} \lambda_{0}\left( t \right)\exp\left( -\Lambda(t;\alpha x_{K}+\sqrt{1-\alpha^{2}}x_{U}) \right)exp(\sigma_{P}\left( t \right)\left( \alpha x_{K}+\sqrt{1-\alpha^{2}}x_{U} \right))\phi\left( x_{U} \right)dx_{U}}{\int_{x_{U}} \exp\left( -\Lambda(t;\alpha x_{K}+\sqrt{1-\alpha^{2}}x_{U}) \right)\phi\left( x_{U} \right)dx_{U}}$$

Under the rare disease assumption, the cumulative rate is assumed to be low, that is $\Lambda(t;x_{P})\approx0$ for all *t* and $x_{P}$. The above expression then simplifies to:

$$\int_{x_{U}} \lambda_{0}\left( t \right)exp(\sigma_{P}\left( t \right)\left( \alpha x_{K}+\sqrt{1-\alpha^{2}}x_{U} \right))\phi\left( x_{U} \right)dx_{U}$$

$${=\lambda}_{0}\left( t \right)exp(\sigma_{P}\left( t \right)\alpha x_{K}+\frac{{\sigma_{P}\left( t \right)}^{2}(1-\alpha^{2})}{2})$$

Thus the marginal log-hazard-ratio associated with the standardised PRS $x_{K}$ is approximately $\sigma_{P}\left( t \right)\alpha=\sigma_{K}\left( t \right)$, as required.

Essentially, since the known and unknown components $x_{K}$ and $x_{U}$are assumed to act multiplicatively on the age-specific incidence, under the rare disease assumption the unknown component can be ignored.

By the same argument, under these conditions the marginal age-specific log-hazard ratio is identical to the conditional log-hazard-ratio given other risk factors (providing that the polygenic component is independent of lifestyle risk factors, as is assumed in BOADICEA),so that other risk factors can be ignored in these derivation. Under these conditions the log-hazard ratio will also be approximately equal to the log-odds-ratio estimated in case-control studies (in practice, these are indeed observed to be similar)^1^.

1. Mavaddat N, Michailidou K, Dennis J, Lush M, Fachal L, Lee A, et al. Polygenic Risk Scores for Prediction of Breast Cancer and Breast Cancer Subtypes. *Am J Hum Genet*. **2019**;104:21-34.
