# Supplementary material for: Incorporating alternative Polygenic Risk Scores into the BOADICEA breast cancer risk prediction model
Source: Cancer Epidemiol Biomarkers Prev. Author manuscript; Available in PMC 2023 Mar 7. (PMC9986688; doi:10.1158/1055-9965.EPI-22-0756)
Supplement: Table S1 [file EMS162586-supplement-Table_S1.docx]

**Supplementary Table S1. Studies and samples used in these analyses**

| **Validation set** | | | **Prospective set** | | |
| --- | --- | --- | --- | --- | --- |
| **Study** | **Controls** | **Cases** | **Study** | **Controls** | **Cases** |
| ABCTB | 71 | 173 | AHS | 1132 | 489 |
| BBCC | 49 | 64 | EPIC | 3597 | 3408 |
| BCEES | 165 | 132 | FHRISK | 0 | 102 |
| BCINIS | 123 | 235 | KARMA | 2986 | 480 |
| BREOGAN | 143 | 237 | NHS | 1660 | 1039 |
| CBCS | 163 | 109 | NHS2 | 1905 | 1112 |
| CCGP | 54 | 130 | PLCO | 2595 | 1725 |
| CGPS | 124 | 207 | PROCAS | 1656 | 342 |
| CPSII | 605 | 340 | SISTER | 1556 | 1494 |
| CTS | 115 | 218 | UKBGS | 701 | 1034 |
| GENICA | 56 | 91 |  |  |  |
| HABCS | 172 | 143 |  |  |  |
| KARMA | 598 | 192 |  |  |  |
| LMBC | 86 | 156 |  |  |  |
| MCBCS | 31 | 94 |  |  |  |
| MCCS | 135 | 76 |  |  |  |
| MISS | 301 | 82 |  |  |  |
| MMHS | 311 | 48 |  |  |  |
| NBHS | 122 | 76 |  |  |  |
| ORIGO | 0 | 144 |  |  |  |
| PBCS | 331 | 217 |  |  |  |
| SEARCH | 193 | 628 |  |  |  |
| SMC | 135 | 226 |  |  |  |
| UCIBCS | 51 | 61 |  |  |  |
| WHI | 845 | 847 |  |  |  |
| Total | 4979 | 4926 |  | 17788 | 11225 |

This is a subset of the samples used in Mavaddat et al. 2019 without missing age at diagnosis of interview and age less than 80 years. This data-set is independent of data used as the training set for the development of PRS313
