# Supplementary material for: Incorporating alternative Polygenic Risk Scores into the BOADICEA breast cancer risk prediction model
Source: Cancer Epidemiol Biomarkers Prev. Author manuscript; Available in PMC 2023 Mar 7. (PMC9986688; doi:10.1158/1055-9965.EPI-22-0756)
Supplement: Table S2 [file EMS162586-supplement-Table_S2.docx]

**Supplementary Table S2. Country in which the studies were conducted**

| **Country** | **Controls** | **Cases** |
| --- | --- | --- |
| Australia | 371 | 381 |
| Belgium | 86 | 156 |
| Canada | 163 | 109 |
| Denmark | 124 | 207 |
| France | 367 | 378 |
| Germany | 922 | 884 |
| Greece | 234 | 302 |
| Israel | 123 | 235 |
| Italy | 780 | 741 |
| Netherlands | 660 | 768 |
| Poland | 331 | 217 |
| Spain | 449 | 536 |
| Sweden | 4020 | 980 |
| UK | 3209 | 2714 |
| USA | 10928 | 7543 |

This is a subset of the samples used in Mavaddat et al. 2019 without missing age at diagnosis of interview and age less than 80 years. This data-set is independent of data used as the training set for the development of PRS313
